# The deregulation of STIM1 and store operative calcium entry impaired aortic smooth muscle cells contractility in aortic medial degeneration

**DOI:** 10.1042/BSR20181504

**Published:** 2019-01-03

**Authors:** Junmou Hong, Zhipeng Hu, Qi Wu, Chaoliang Tang, Junxia Hu, Ruoshi Chen, Bowen Li, Zhiwei Wang

**Affiliations:** 1 Department of Cardiothoracic Surgery, Renmin Hospital of Wuhan University, Wuhan, Hubei Province, China; 2 Department of Central Laboratory, Renmin Hospital of Wuhan University, Wuhan, Hubei Province, China; 3 Department of Anesthesiology, The First Affiliated Hospital of USTC, Division of Life Sciences and Medicine, University of Science and Technology of China, Hefei, Anhui Provence, China

**Keywords:** aortic smooth muscle cells, endoplasmic reticulum stress, STIM1, transforming growth factor β1

## Abstract

**Background:** Microarray analysis of clinical aortic samples suggested a potential role for stromal interaction molecule 1 (STIM1) in the modulation of aortic medial degeneration (AMD), despite the uncertainty about STIM1 in normal aortic smooth muscle cells (ASMCs). Here, we aimed to explore changes in STIM1 expression in AMD, and the possible mechanisms. **Methods:** An AMD model was established using auto-delivery of angiotensin II (Ang II) into ApoE^−/−^ mice. We assessed the effects of SKF96365, a STIM1 inhibitor, in AMD model and *in vitro* cultured ASMCs. Elastic van Gieson (EVG) staining was used to visualize elastic fiber injury. Mitochondria changes were viewed by TEM. Cytoplasmic calcium was quantified by measuring fluo-4 staining in a flow cytometer. Mechanical stretching device was used to mimic stretching that ASMCs experience *in vivo*. Cell apoptosis was determined by using Annexin V/propidium iodide (PI) staining. The expression of STIM1, contractile related proteins (α-smooth muscle actin (α-SMA), myosin light chain (MLC)), endoplasmic reticulum (ER) stress-related proteins (CHOP, activating transcription factor 6 (ATF-6)) and smad2/3 were assessed by Western blotting, immunohistochemistry (IHC), and immunofluorescence (IF). **Results:** SKF96365 exacerbated aortic injury in the AMD model. SKF96365 reduced cytoplasmic calcium concentration in ASMCs, caused mitochondrial swelling, and elevated the expression of ATF-6 and CHOP. SKF96365 decreased the expression of MLC and α-SMA in ASMCs, causing them to be vulnerable to mechanical stretch. SKF96365 suppressed smad2/3 activation after treatment with transforming growth factor (TGF) β1 (TGFβ1). **Conclusions:** STIM1 is indispensable in ASMCs. Interfering with STIM1 exaggerated the AMD process by modulating the expression of contractile proteins, inducing ER stress in ASMCs.

## Introduction

Aortic medial degeneration (AMD), characterized both by the loss of aortic smooth muscle cells (ASMCs) and phenotypic modulation, is a common pathological feature in aortic dissection (AD) and aortic aneurysm (AA). Phenotypic modulation occurs when quiescent and contractile ASMCs are transformed to a proliferative, synthetic phenotype. ASMCs normally exhibit strong contractile characteristics to withstand powerful blood flow. Impairment of contractile capacity leads to aortic dilation and possibly rupture. Patients with mutant genes that are associated with contraction, such as MYH11, ACTA2, or MYLK are susceptible to hereditary AD [1]. However, for other patients, the reasons for their ASMCs to exhibit degenerative contractile capacity remains unclear.

Calcium is a key element in the excitation contraction coupling process of ASMCs in addition to various contractile proteins. Vitamin D deficiency induced deregulated calcium is a hallmark of thoracic aortic dilatation and aneurysm in both patients and established AD animal models [2,3]. Stress on the endoplasmic reticulum (ER), a cell’s internal resource of calcium, also plays an important role in establishing an AD model [4,5]. Under normal circumstances, ASMCs exhibit two phases in elevated cytosolic calcium concentration following stimulation. The initial transient increase is due to inositol trisphosphate (IP_3_)-mediated release of ER calcium. Evidence has shown that the contractile capacity of ASMCs is significantly suppressed in IP_3_R1-deficient aortas [6]. A subsequent prolonged increase in calcium requires an influx of extracellular ions through activation of voltage-operated or receptor-operated calcium channels. Stromal interaction molecule 1 (STIM1) is an ER calcium sensor which activates plasma membrane calcium channels in conditions where the ER is depleted of calcium. Calcium depletion induces STIM1 to oligomerize and move to the ER/plasma membrane junctional domains, where it interacts with and activates the plasma membrane calcium channels. This process has been documented as store-operated calcium entry (SOCE).

Previous reports have indicated that STIM1 has no apparent role in normal ASMCs [7], but recent microarray analysis of clinical samples has provided intriguing evidence that STIM1 may be a factor in AMD [8]. Evidence suggests that vitamin D deficiency suppresses the expression of a number of calcium-regulating proteins, including STIM1 [9,10]. As is well known, AMD is closely related to the process of ageing. It has been shown that tension in the mesenteric artery is reversed and patterns of STIM1 and Orail expression change with ageing [11]. Taken together, we hypothesize that down-regulation of STIM1 may cause AMD.

In the present study, we investigated STIM1 expression in human aortic samples. We further assessed the role of SKF96365, a SOCE inhibitor, in the regulation of AMD progression in an established AD mouse model. Interference of SOCE exacerbated the aortic injury in the AD mouse model by modulating the expression of contractile proteins, causing stress in the ER and cytoskeleton of ASMCs.

## Materials and methods

### AD mouse model

The Ethical Committee of Renmin Hospital of Wuhan University approved the animal study protocol. The handling of all animals was performed in accordance with the Wuhan Directive for Animal Research and Current Guidelines for the Care and Use of Laboratory Animals published by the National Institutes of Health. Male ApoE^−/−^ mice (C57BL/6J background) were obtained from the Department of Laboratory Animal Science (Peking University Health Science Center, Beijing, China). Six-week-old male mice were implanted with Alzet osmotic mini-pumps (Model 2004, Durect Corporation, U.S.A.), filled with either saline or angiotensin II (Ang II) solution (1 mg/kg/min) for up to 4 weeks. In a preliminary assessment of treatment with SKF96365 (S7999, Selleck Corporation, U.S.A.) *in vivo*, 12 ApoE^−/−^ mice without osmotic mini-pumps were divided into two groups (*n*=6 per group) and then administered with either saline or SKF96365 (20 mg/kg) by intraperitoneal injection (ip) each day for a week. In the formal experiment, 36 ApoE^−/−^ mice were divided into three groups (*n*=12 per group), NC (no treatment) group, Ang II pumping + saline ip (Ang II + saline group), and Ang II pumping + SKF96365 ip (Ang II + SKF96 group).

### Histopathology, immunohistochemistry, and TEM

Details of the clinical aortic samples that were used in this study are summarised in Supplementary Table S1. Aortic samples were fixed in 4% formaldehyde overnight, dehydrated, paraffin-embedded, and then sectioned. The sections were stained with Masson’s trichrome or Elastic van Gieson (EVG) stain using previously published protocols [12,13].

Immunohistochemistry (IHC) was performed as previously reported [14]. Briefly, the sections were deparaffinized in xylene, rehydrated, and endogenous peroxidase inactivated by incubation in 3% hydrogen peroxide for 10 min at 37°C. Antigen retrieval was achieved by heating sections submersed in 10 mM sodium citrate in a microwave for 15 min. Non-specific antibody binding was blocked by incubating the slides in PBS containing 5% goat serum for 1 h at 37°C. The slides were then stained with a panel of primary antibodies (STIM1, phosphorylated-myosin light chain (p-MLC), α-smooth muscle actin (α-SMA), F-actin, and smad2/3) overnight at 4°C and then processed using a streptavidin–biotin complex peroxidase (mouse/rabbit IgG) kit (SA1020, Boster Corporation) according to the manufacturer’s instructions. Details of the antibodies used are given in Supplementary Table S2. Visualization of positive staining was possible after developing with 3,3′-diaminobenzidine (DAB). Tissue structure was visible after subsequent counterstaining with Hematoxylin. The grading of positive staining was in accordance with a previously published report [15].

Cellular ultrastructure was observed by TEM (H-7000 FA, Hitachi, Japan). This analysis was performed by Servicebio Co. (Wuhan, China).

### Cell culture and treatment

The human ASMC (H-ASMC) line (ATCC® PCS-100-012™) was purchased from the China Center for Type Culture Collection (CCTCC) and cultured in H-ASMC complete medium (Cat# CM-H081, Procell, China) at 37°C in an atmosphere of 5% CO_2_ and 100% humidity. SOCE inhibition was achieved by adding SKF96365 to cells at concentrations up to 50 μM.

Stimulation with mechanical stretch was performed as described in detail previously [16]. Briefly H-ASMCs were pre-treated with SKF96365 or DMSO for 6 h followed by mechanical strain (2333 μm, Strain; 50  Hz) for 2 or 4 h prior to harvesting for analytical experiments (model diagram in Figure 4A).

To investigate the effect of SOCE on transforming growth factor (TGF) β1 (TGFβ1)/Smads signaling, H-ASMCs were pre-treated with saline or SKF96365 for 6 h prior to administration with TGFβ1 (5  ng/ml) for 30, 60, or 120 min. The cells were then harvested for subsequent analysis.

### Cell viability and apoptosis assays

Cell Counting Kit-8 (MA0218, Dalian Meilune Biotechnology Corporation, China) was used to assess the influence of SKF96365 on cell viability. H-ASMCs were seeded in 96-well plates, 5 × 10^3^ cells per well in 180 μl culture medium, then administrated with SKF96365 (10 μl) at doses up to 50 mM for 24 h. Subsequently, 10 μl of WST-8 was added to each well and the plates were incubated for a further 4 h. Absorbance at 450 nm was measured using a microplate reader (PerkinElmer Victor 1420, U.S.A.).

Cell apoptosis was evaluated using an Annexin V/propidium iodide (PI) double staining kit (70-AP101-60, MultiSciences Corporation, China), in which cells of interest were suspended in 500 μl PBS containing 2 μl Annexin V and 2μl PI for 30 min prior to analysis by flow cytometry (BD FACS Calibur, U.S.A.).

## Western blotting

To analyze the distribution of protein expression after the various cellular treatments described above, total protein was extracted from H-ASMCs and aortic samples using RIPA buffer (P0013B, Beyotime Corporation, China) containing a cocktail of the following protease inhibitors (Roche 4693159001, Merck Corporation, U.S.A.) and PMSF (MA0001, Dalian Meilune Biotechnology Corporation, China). Protein concentration was quantitated using a BCA assay (P0011, Beyotime Corporation, China). Equal quantities of samples underwent protein denaturation, followed by 8–12% SDS/PAGE with transfer of the separated proteins on to PVDF membranes. The membranes were incubated in PBS containing 5% skim milk to block non-specific binding and then overnight at 4°C in a solution containing primary antibody (STIM1, α-SMA, MLC, activating transcription factor 6 (ATF-6), CHOP, smad2/3, p-smad2/3, GAPDH). Each membrane was then stained with a secondary antibody and positive binding visualized using an Odyssey infrared imager (LI-COR, Nebraska, U.S.A.), the gray scale of each band was determined by the device software. Details of all antibodies used are shown in Supplementary Table S2.

### Immunofluorescence imaging

H-ASMCs were plated on coverslip, and treated as previouly. Then cells were fixed in 4% formaldehyde for 1 h and incubated with PBS containing 0.1% Triton X-100 to increase cellular membrane permeability. Non-specific binding was blocked using 5% goat serum prior to incubating the cells with primary antibodies overnight at 4°C. Cells were then incubated with a secondary antibody conjugated with a fluorescent label (Cy3 or FITC) for 1 h at room temperature and the cell nuclei counterstained with DAPI. Images were captured using a confocal microscope (FV1200, Olympus, Japan) or fluorescence microscope (BX63, Olympus, Japan). Details of the antibodies used are shown in Supplementary Table S2. Quantitation of the results was achieved by measuring fluorescence in five randomly selected fields in each slide using ImageJ software (Rawak Software, Inc., Germany).

### Cellular migration assays (Transwell assays)

Transwell chambers were purchased from Corning. Into each apical chamber, 5 × 10^4^ cells in a total volume of 180 μl serum-free medium contained saline or SKF96365 (0.4, 2, or 10 μM) were placed. Eight hundred microliters of complete medium was placed into the basal chamber. After incubation for 24 h, the cells in the apical chamber were stretched and then stained with 0.25% Crystal Violet. Images were captured using a light microscope (BX51, Olympus, Japan). Cells were counted at a magnification of ×400 in five randomly selected fields from each chamber.

### Measurement of cytoplasm calcium concentration

Cells were collected and then incubated in PBS contained fluo-4 AM (1 μM) for 30 min at room temperature. They were washed twice in PBS and further incubated for 30 min. Cellular fluorescence at excitation and emission frequencies of 495 nm and 516 nm, respectively, was measured using flow cytometry (BD FACS Calibur, U.S.A.).

## Statistical analysis

All quantitative data are presented as mean ± S.D. Statistical analysis was performed using GraphPad Prism 5 software (GraphPad Software, U.S.A.). Quantitative data were analyzed using Student’s *t*test (two-tailed) or one-way ANOVA. *P*<0.05 was defined as being statistically significant.

## Results

### Treatment with SKF96365 reduced expression of STIM1, impaired mitochondria, and caused ER stress in ASMCs

In the initial part of the study, physiological changes in the aortic samples were evaluated. Consistent with previous studies, we observed a significant decrease in the expression of p-MLC and α-SMA in the AD group compared with that of the donor group (Supplementary Figure S1A). Masson’s trichrome staining demonstrated a decrease in numbers of muscle fibers in the AD samples (Supplementary Figure S1B). IHC staining indicated down-regulation of STIM1 in the AD samples (Supplementary Figure S1B) with similar results when Western blotting was used to examine the expression of STIM1 and α-SMA (Supplementary Figure S1D). To confirm our findings, we cross-referenced the expression levels of STIM1 with two GEO databases (GEO: GSE52093, AD database; GEO: GSE57691, AA database), and found similar results (Supplementary Figure S1C). To further explore possible mechanisms, SKF96365 was used to modify the function of SOCE.

**Figure 1:**
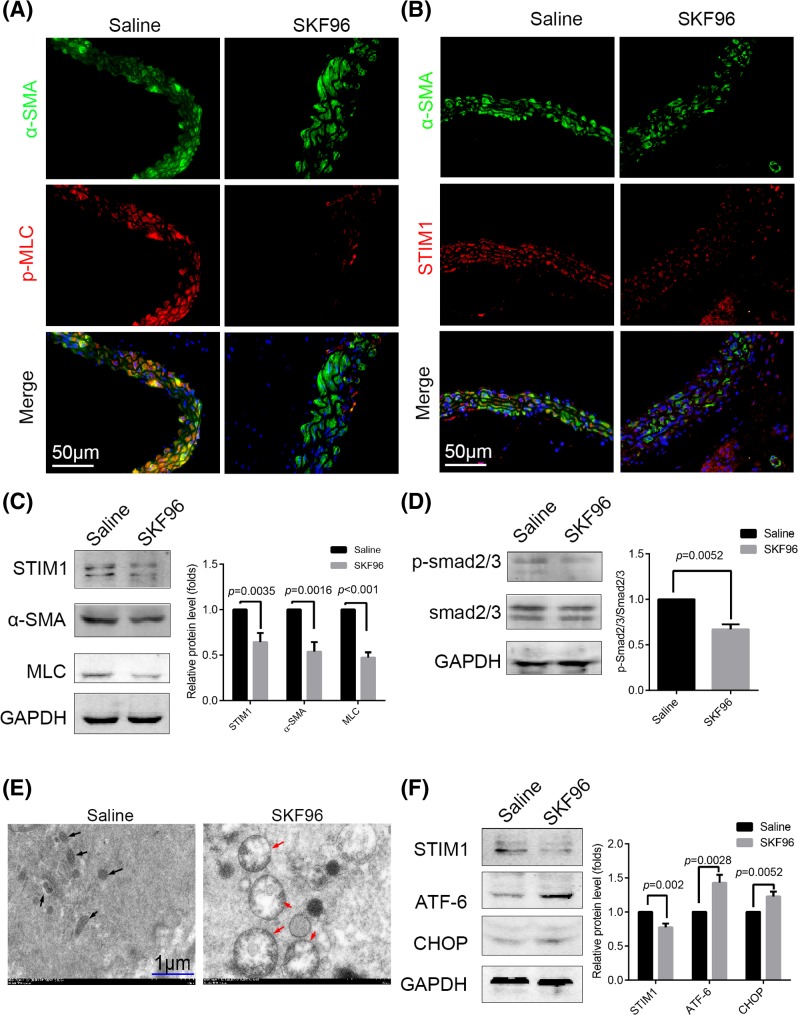
SKF96365 reduced STIM1 expression, impaired mitochondria, and led to ER stress in ASMCs *in vivo* ApoE^−/−^ mice were treated with SKF96365 (*n*=6) or saline (*n*=6) by intraperitoneal injection for 1 week. (**A**,**B**) Immunofluorescence images of p-MLC and STIM1 in ASMCs (marked by α-SMA staining) after SKF96365 treatment for 1 week; *n*=6. (**C**) Presence of STIM1, α-SMA, and MLC as determined in SKF96 and control group aortic samples. (**D**) Phosphorylation of smad2/3 and expression of total smad2/3 assessed by Western blotting in the two groups. (**E**) ASMCs of the SKF96 group exhibited apparent swelling and deformation of the mitochondria (red arrows) compared with the saline group (black arrows), as observed by TEM. (**F**) Expression of the ER stress-related proteins ATF-6 and CHOP was elevated in the aortic samples of SKF96365-treated mice by Western blotting. All Western blotting analyses were performed three times. Representative images are shown. Data are represented as mean ± S.D.(*t test, two-sided).*

First, the effects of injection of SKF96365 into ApoE^−/−^ mice were examined. After 1 week p-MLC and STIM1 expression in ASMCs (marked by α-SMA staining) decreased (Figure 1A,B). Furthermore, we used Western blotting to evaluate the expression of the contractile proteins (α-SMA and MLC) and found that both were reduced in the SKF96365 group (Figure 1C). To further ascertain the importance of TGFβ1-smad2/3 signaling in AD and AA, we measured the expression of p-smad2/3, revealing that SKF96365 decreased smad2/3 activation (Figure 1D). We also wished to determine the importance of STIM1 in maintaining stable ER function. TEM was used to observe ultrastructural changes in ASMCs. Clearly swollen mitochondria were observed in the ASMCs of the SKF96365 group, although the ER can hardly be seen (Figure 1E). We speculated that SKF96365 might perform a role in potentiating stress in the ER. Thus, we investigated related ER stress protein level expression and observed elevated levels of ATF-6 and CHOP using Western blotting (Figure 1F).

### SKF96365 intensified the severity of AMD in an established mouse model of AD

**Figure 2:**
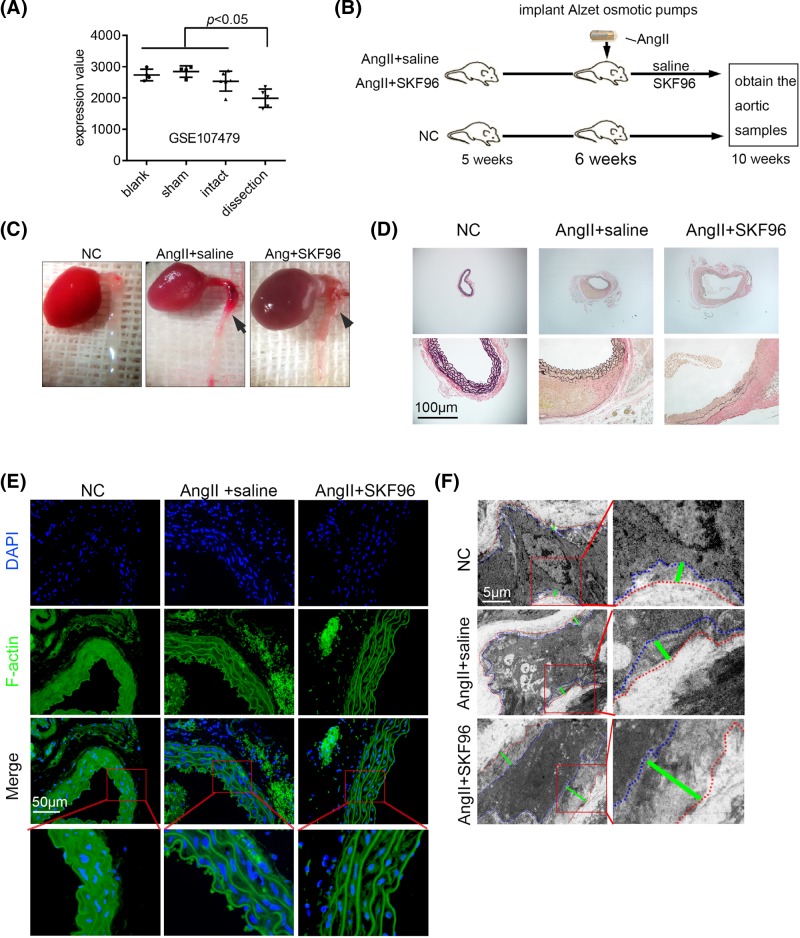
SKF96365 exacerbated aortic injury in an established AD mouse model (**A**) STIM1 expression in a dataset (GEO: GSE107479) of established AD, induced by application of 0.5 M CaCl_2_ to the infrarenal aorta and continuous infusion of AngII (1 mg/kg/min) in wild-type mice. (**B**) Experimental flow chart (*n*=12 per group). (**C**) General views of thoracic aortas are shown. The black arrows show aortic lesions. (**D**) EVG staining showing breakage of elastic fibers of aortic samples; *n*=4. (**E**) F-actin staining of remodeled cytoskeletal and significant shrinkage of ASMCs in the AngII+SKF96 group; *n*=6. (**F**) TEM images showing the distance between the plasma membrane and elastic fibers. The ASMC plasma membrane boundary (blue dotted line), elastic fiber boundary (red dotted line), and the distance between them (green line) are shown; *n*=3.

After confirming the suitability of using SKF96365 in the *in vivo* model, we aimed to explore the influence of SOCE on AD. We confirmed our hypothesis that STIM1 decreases in AD by re-analyzing the dataset from an established AD model (GEO: GSE107479) (Figure 2A). A flow chart depicting the experimental design of these experiments is shown in Figure 2B. Images of samples of mouse model thoracic aortas with AD are shown in Figure 2C. The aortic specimens were stained with EVG to show elastic fibers. Aortic dilation and elastic fiber breakage were more apparent in the AngII + SKF96 group than in the AngII + saline group (Figure 2D). The powerful effects of AngII on the control of morphology and cytoskeleton in ASMCs, which was stained using phalloidin to target F-actin, can easily be seen. Shrinkage of ASMCs was apparent in the AngII + SKF96 group (Figure 2E), a result confirmed by TEM (Figure 2F).

### SKF96365 suppressed ASMC viability and mobility

**Figure 3:**
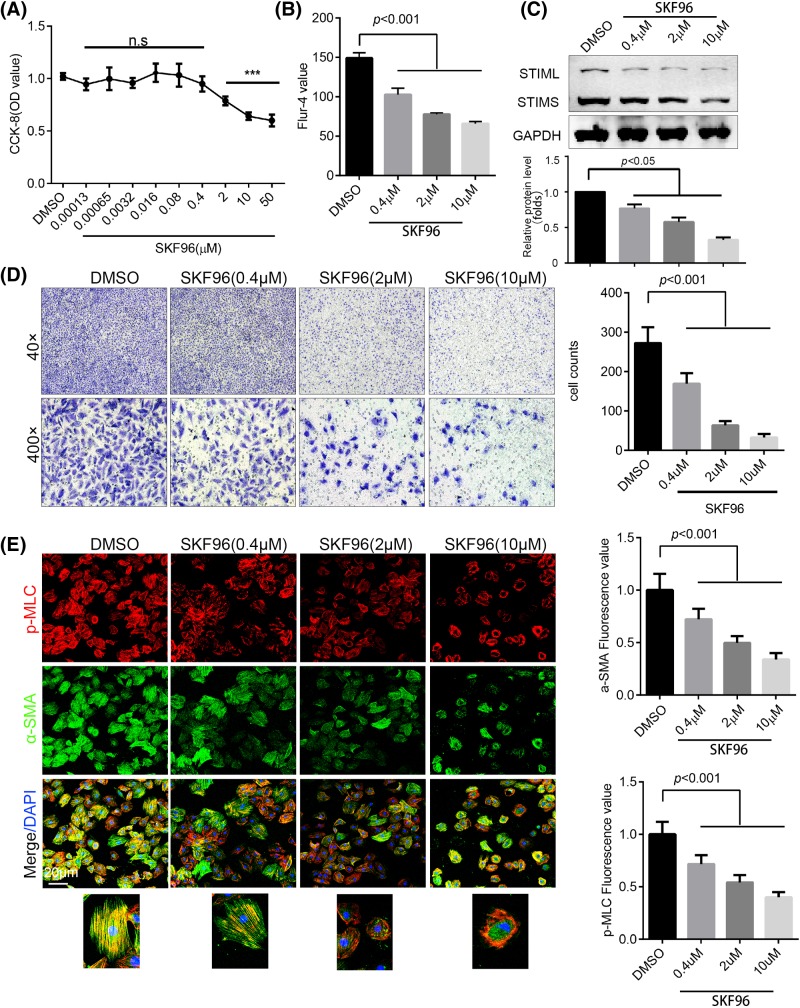
SKF96365 impaired H-ASMC viability and mobility (**A**) Cell viability after treatment with different doses of SKF96365 for 24 h, as assessed by CCK-8 assay; *n*=6. (**B**) Cytoplasmic calcium was stained by Fluo-4 and detected by flow cytometry; *n*=3. (**C**) H-ASMCs treated with different doses of SKF96365 for 24 h, with STIM1 expression detected by Western blotting; *n*=3. (**D**) H-ASMCs pre-treated with different doses of SKF96365 for 6 h, with migration detected using a transwell assay. Representative images are shown; *n*=3. (**E**) H-ASMCs administrated with various doses of SKF96365 for 24 h. p-MLC (red) and α-SMA (green) were stained and images captured by confocal microscopy (left panel). The fluorescence value was calculated by ImageJ; *n*=3. Data are represented as mean ± S.D., ***P<0.001 compared with DMSO group.

To elucidate the mechanism of SOCE-mediated injury in ASMCs, an H-ASMC cell line was used to assess the effect of SKF96365 on ASMCs *in vitro*. We found that SKF96365 injured the viability of H-ASMCs in a dose-dependent manner (Figure 3A). We further demonstrated that SKF96365 down-regulated cytosolic calcium concentration using a flow cytometer (Figure 3B). Moreover, SKF96365 decreased the expression of STIM1 in a dose-dependent manner (Figure 3C). Cell mobility was assessed using a transwell assay and revealed that SKF96365 significantly reduced the capacity of H-ASMCs to migrate, even at doses (0.4 μM) that had no obvious cytotoxicity (Figure 3D). To explore the change in p-MLC and α-SMA expression after SKF96365 administration, H-ASMCs were visualized by confocal microscopy. Treatment with SKF96365 led to a decrease in p-MLC and α-SMA in a dose-dependent manner. In addition, SKF96365 promoted the translocation of p-MLC to a site close to the cell membrane (Figure 3E). In addition to SOCE, SKF96365 is able to inhibit voltage-gated calcium and potassium channels. Thus, we constructed siRNA targetted to STIM1 (si-STIM1). Consistent with SKF96365, knockdown STIM1 using si-STIM1 decreased cytosolic calcium concentration (Supplementary Figure S2B). Knockdown of STIM1 also decreased the expression of MLC and α-SMA (Supplementary Figure S2A), and promoted the translocation of p-MLC be closed to the cell membrane (Supplementary Figure S2C).

### Low-dose SKF96365 intensified H-ASMC injury when mechanically stretched

**Figure 4:**
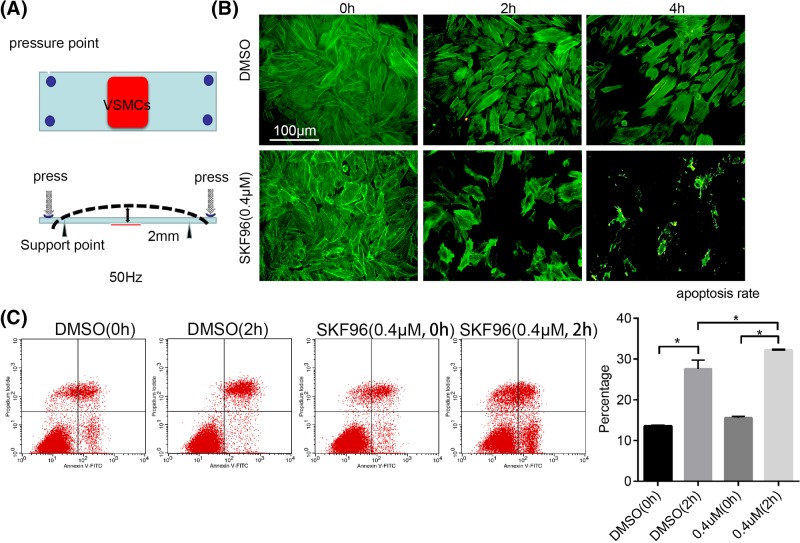
Low-dose SKF96365 exacerbated injury to H-ASMCs under mechanical stretch (**A**) Schematic representation of mechanical stretch is shown. (**B**) H-ASMCs pretreated with SKF96365 (0.4 μM) for 6 h then mechanically stretched for 2 or 4 h. F-actin stained by phalloidin; *n*=3. (**C**) Apoptosis of H-ASMCs after receiving SKF96365 and mechanical stretching assessed by Annexin V/PI double staining; *n*=3. Data represented as mean ± S.D. (*t test, two-sided*). *, *P*<0.05.

To mimic the experience of ASMCs *in vivo*, a mechanical stretch device was utilized [16]. A schematic representation is shown in Figure 4A. H-ASMCs were seeded on a special plate (red area) as indicated. Pressure was applied at four points to bend the plate, causing the attached cells to overstretch. Experimental cells were fixed and stained with phalloidin. Pretreatment with SKF96365 (0.4 μM) for 6 h caused H-ASMCs to die when mechanically stretched for 2 or 4 h (Figure 4B). The cells still attached to the plate were resuspended and analyzed using Annexin V/PI double staining. H-ASMCs pre-treated with SKF96365 exhibited significantly elevated apoptosis (Figure 4C).

### SKF96365 suppressed smad2/3 activation and contractile-related protein expression, leading to ER stress

**Figure 5:**
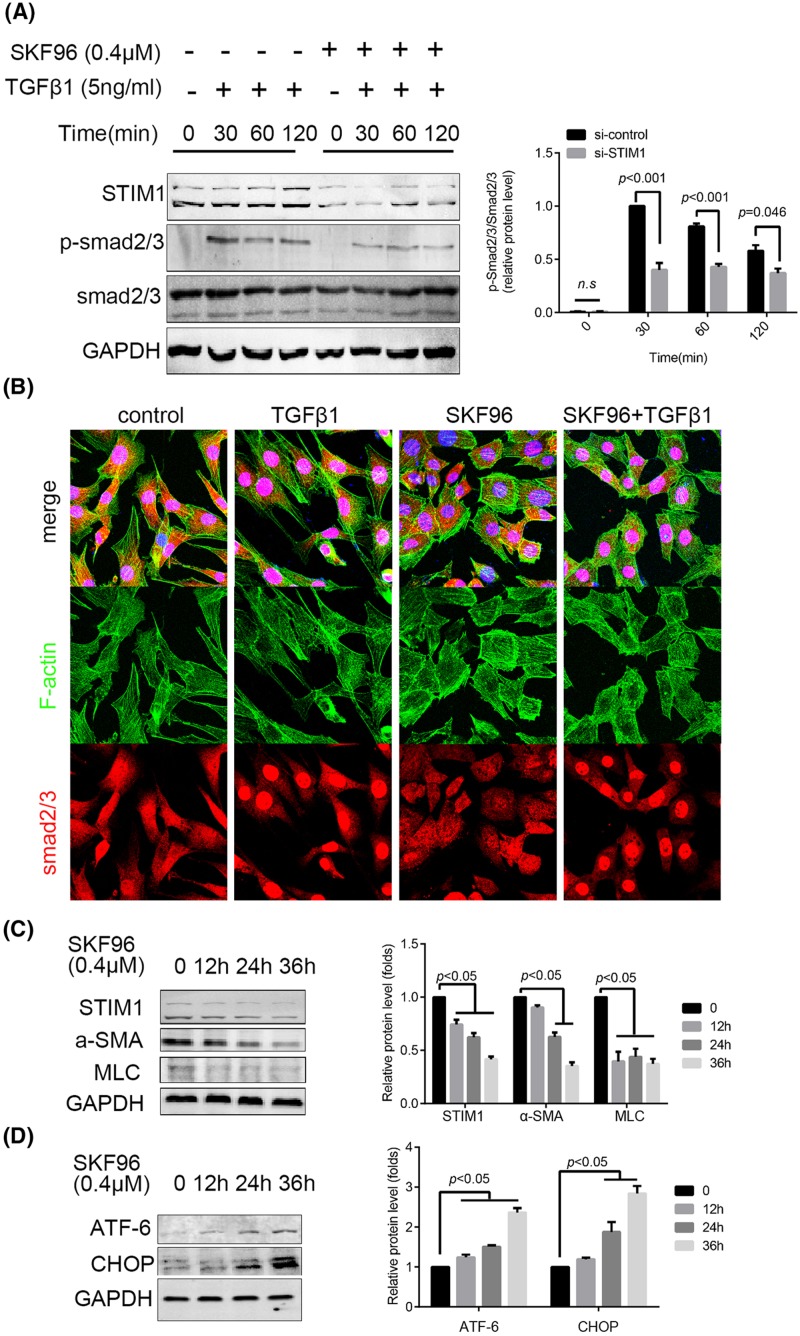
SKF96365 suppressed smad2/3 activation, contractile-related protein expression, leading to ER stress (**A**) H-ASMCs were pretreated with SKF96365 (0.4 μM) for 6 h followed by administration of TGFβ1 (5 ng/ml) over various times. p-smad2/3 and total smad2/3 were detected by Western blotting; *n*=3. (**B**) H-ASMCs were pre-treated with SKF96365 followed by TGFβ1 (5 ng/ml) for 30 min. F-actin and smad2/3 were stained. Images were captured by confocal microscopy; *n*=3. (**C**) SKF96365 suppressed α-SMA and MLC expression in a time-dependent manner. (**D**) SKF96365 elevated expression of ATF-6 and CHOP in a time-dependent manner; *n*=3. Data represented as mean ± S.D. (*t test, two-sided*).

TGFβ1-smad2/3 signaling is important for maintenance of the contractile phenotype of ASMCs [17]. Thus, we explored the influence of SKF96365 on smad2/3 activation after TGFβ1 simulation by Western blotting. The results demonstrated that SKF96365 significantly reduced the phosphorylation of smad2/3 (Figure 5A). The translocation of smad2/3 to the nucleus induced by TGFβ1 was suppressed by SKF96365 pretreatment (Figure 5B). Even though low doses of SKF96365 did not exhibit apparent cytotoxicity, it still decreased the expression of α-SMA and MLC (Figure 5C). Consistent with the results of the *in vivo* experiment, SKF96365 was effective in elevating the expression of ATF-6 and CHOP (Figure 5D). The expression of ATF-6 and CHOP were also elevated in si-STIM1 cells (Supplementary Figure S3).

## Discussion

During the progression of AMD, ASMCs mainly exhibit decreased contractility and apoptosis [18]. These two characteristics are linked *in vivo*. A decrease in contractility results in ASMC overstretch when experiencing the powerful impact of blood flow. This overstretch may cause damage to the cells and lead to cell death [19]. A decrease in ASMC numbers will, in turn, exacerbate the stress experienced by surviving cells, creating a vicious circle. At present, the cause of reduced smooth muscle contractility is not clear. The present study confirmed that the reduction in STIM1 led to a reduction in smooth muscle contraction-related protein expression and induced stress in the ER.

Previous studies have suggested that the function of SOCE in freshly isolated ASMCs is weak [7,20]. Recently, research by Albert et al. has demonstrated that in contractile vascular SMCs, calcium storage depletion can result in ER remodeling, promotion of STIM1-TRPC1/Orai1 interaction and induction of calcium channel opening [21]. These results suggest that SOCE may be dispensable in ASMCs. In clinical practice, it can be observed that the progression of AMD is closely related to gender and ageing [22]. Changes in SOCE function exhibit similar gender differences and is age-related [11,23]. These two observations may be relevant. According to Butt et al. [8], STIM1 is one of three statistically significant down-regulated genes in AA. Our results and microarray re-analysis demonstrated a significant decrease in STIM1 in patients with AD and AA. These observations suggest that SOCE also performs an important role in regulating the function of ASMCs during the progression of AMD.

Inactivation of MLC by mutation of its CaM-binding site leads to hereditary AD [24]. Our study showed that p-MLC decreased in AD aortic samples. A significant decrease in the expression of p-MLC was observed after the inhibition of SOCE in the mouse model. Inhibition of SOCE decreased calcium concentration in the cytoplasm, suppressed activation of CaM resulting in MLC inactivation. Our research demonstrated that high doses of SKF96365 impairs cell viability. Low doses, although not significantly influencing cell viability alone, render ASMCs vulnerable to mechanical stretch, possibly resulting from its suppression of MLC activation.

A recent study demonstrated that a specific knockout of polo-like kinase 1 (PLK1) in ASMCs led to spontaneous AD through remodeling of the cytoskeleton [25]. The ASMCs exhibited a round morphology after ablation of PLK1 [25]. In our study, ASMCs demonstrated a similar change when treated with SKF96365. Mice administered with SKF96365 exhibited significant shrinkage of ASMCs. The *in vitro* study also demonstrated that inhibiton of SOCE by treatment with SKF96365 caused ASMCs to become more round with fewer actin fibers.

As STIM1 is a calcium sensor on the ER, any abnormality in its function is inevitably linked to stress of the ER. Studies have shown that ER stress contributes to AMD [4]. At present, the way in which STIM1 causes ER stress is still controversial. Interference of STIM1 function has been shown to alleviate ER stress in some experiments [26]. However, in animal experiments, STIM1 knockout resulted in significant endoplasmic reticular and mitochondrial dysfunction in the myocardium [27]. In this study, we observed apparently swollen mitochondria in the ASMCs of SKF96365-treated mice using TEM. This suggests that inhibition of SOCE function may lead to ER stress in ASMCs, a result confirmed in further experiments by detection of CHOP and ATF-6 expression in both aortic samples and in *in vitro* experiments. However, the reverse trend of GRP78 expression related to SKF96365 treatment was observed (data not shown). This result should be further studied.

Continuous activation of TGFβ1-smad2/3 signaling is required for maintenance of the contractile phenotype of ASMCs [17]. We found that SKF96365 has the capability to inhibit smad2/3 phosphorylation and nuclear translocation, consistent with the studies of Mai et al. [28]. When TGFβ1 induces differentiation of stem cells into smooth muscle cells, CaMKII regulates SM22a and α-SMA expression [29]. CaMKII potentiates up-regulation of SOCE by promoting STIM1 aggregation [30], possibly the reason for low-dose SKF96365 also leading to a decline in α-SMA and MLC expression without causing significant cytotoxicity.

In recent years, studies have found that STIM1 exhibits two isoforms, STIM1L and STIM1S. Reports have shown STIM1L is responsible for rapid calcium release [31] whereas STIM1S regulates a change in ER morphology [32]. The influence of STIM1 subtype on smooth muscle in AMD remains to be investigated.

In addition to inhibiting SOCE function, SKF96365 can also inhibit voltage-activated calcium and potassium channels. The 50% inhibitory concentration (IC_50_) of SKF96365 was measured as 0.85 µM for ATP-sensitive K^+^ channels (IKATP) and 1 µM for voltage-gated K^+^ channels (IKv) in mouse small intestinal smooth muscle cells. However, SKF96365 (1 µM) had no significant effect on spontaneous transient calcium activated K^+^ channels (IBK) or caffeine-induced IBK [33]; 10 µM of SKF96365 was sufficient to suppress IBK in human airway smooth muscle cells [34]. According to Singh et al. [35], human CaV3.1 T-type Ca channels are more potently inhibited by SKF96365 (IC_50_: 0.56 M) *in vitro*. The lowest SKF96365 concentration used in our experiments (0.4 μM) is close to Singh et al. [35] study (0.56 μM). Given the non-specific effects of SKF96365, it is possible that the contributions toward SOCE might have been overestimated. Thus, we also constructed an shRNA that targetted STIM1. Consistent with SKF96365, STIM1 knockdown decreased cytoplasmic calcium concentration. Moreover, knockdown also resulted in impaired H-ASMC mobility, down-regulation of α-SMA and MLC, and up-regulation of the ER stress-related proteins ATF-6 and CHOP.

In summary, we have demonstrated that decreased STIM1 is involved in the progression of AMD. Inhibiton of SOCE in ASMCs decreased contractile proteins, attenuated smad2/3 activation, remodeled the cytoskeleton, modulated ER stress, and exacerbated aortic injury in an established AD mouse model. These observations prove that decreased levels of STIM1, perhaps because of ageing, is a key mechanism involved in AMD.

## Supporting information

**Supplementary Figure S1:** 

**Supplementary Figure S2:** 

**Supplementary Figure S3:** 

**Table S1.** The clinical characters of the patients that contributed the sample in this study

**Table S2.** The detail of antibodies used in this study

## Abbreviations

AA: aortic aneurysm
AD: aortic dissection
AMD: aortic medial degeneration
Ang II: angiotensin II
ASMC: aortic smooth muscle cell
ATF-6: activating transcription factor 6
ER: endoplasmic reticulum
EVG: Elastic van Gieson
H-ASMC: human ASMC
IHC: immunohistochemistry
MLC: myosin light chain
PI: propidium iodide
PLK1: polo-like kinase 1
SOCE: store-operated calcium entry
STIM1: stromal interaction molecule 1
TGFβ1: transforming growth factor
α-SMA: α-smooth muscle actin
