# Correction: The deregulation of STIM1 and store operative calcium entry (SOCE) impaired aortic smooth muscle cells contractility in aortic medial degeneration

**DOI:** 10.1042/BSR-20181504_COR

**Published:** 2020-07-21

**Keywords:** aortic smooth muscle cells, endoplasmic reticulum stress, STIM1, transforming growth factor β1

The authors of the original paper “The deregulation of STIM1 and store operative calcium entry (SOCE) impaired aortic smooth muscle cells contractility in aortic medial degeneration” (*Biosci Rep.* (2019), **39**(1), DOI: 10.1042/BSR20181504), would like to provide the following correction statement:

The picture in Figure 3D was replaced by a new set of images.

**Figure 3:**
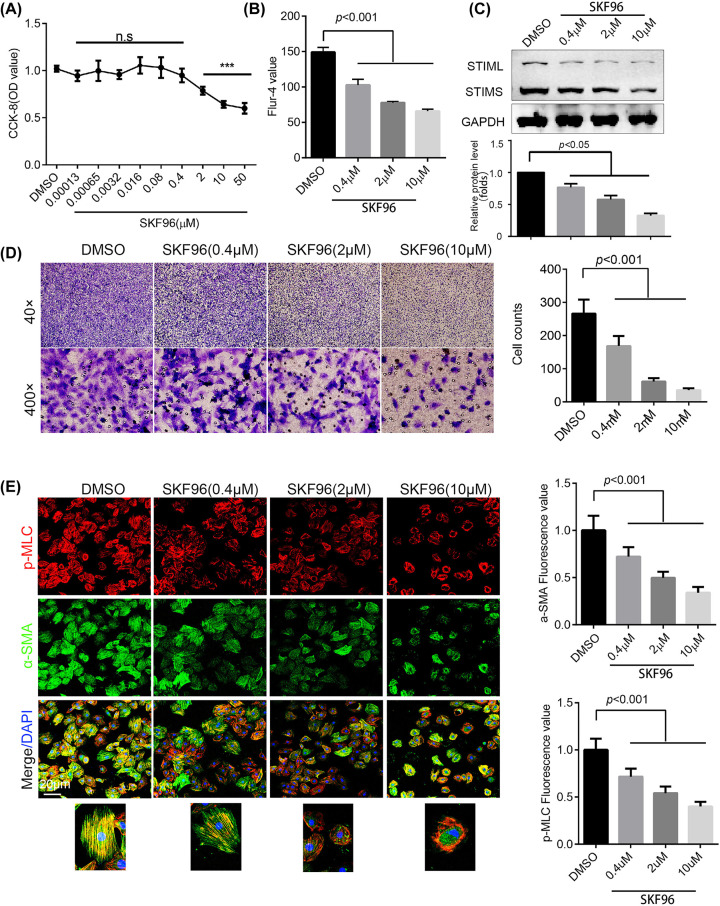
SKF96365 impaired H-ASMC viability and mobility (**A**) Cell viability after treatment with different doses of SKF96365 for 24 h, as assessed by CCK-8 assay; *n* = 6. (**B**) Cytoplasmic calcium was stained by Fluo-4 and detected by flow cytometry; *n* = 3. (**C**) H-ASMCs treated with different doses of SKF96365 for 24 h, with STIM1 expression detected by Western blotting; *n* = 3. (**D**) H-ASMCs pre-treated with different doses of SKF96365 for 6 h, with migration detected using a transwell assay. Representative images are shown; *n* = 3. (**E**) H-ASMCs administrated with various doses of SKF96365 for 24 h. p-MLC (red) and α-SMA (green) were stained and images captured by confocal microscopy (left panel). The fluorescence value was calculated by ImageJ; *n* = 3. Data are represented as mean ± S.D., ***P<0.001 compared with DMSO group.

Due to our carelessness, the image of transwell experiment of 0.4μM (40×) were the magnify of that of DMSO group (100×).

We apologise for this mistake. We have repeated this experiment, and present the correct image in this revised version. We confirm that it does not affect the conclusions of the study.

