# Supplementary material for: The deregulation of STIM1 and store operative calcium entry impaired aortic smooth muscle cells contractility in aortic medial degeneration
Source: Biosci Rep. 2019 Jan 3;39(1):BSR20181504. doi: 10.1042/BSR20181504 (PMC6328863; doi:10.1042/BSR20181504)
Supplement: Supplementary file 1 [file bsr20181504_Supp1.pdf]

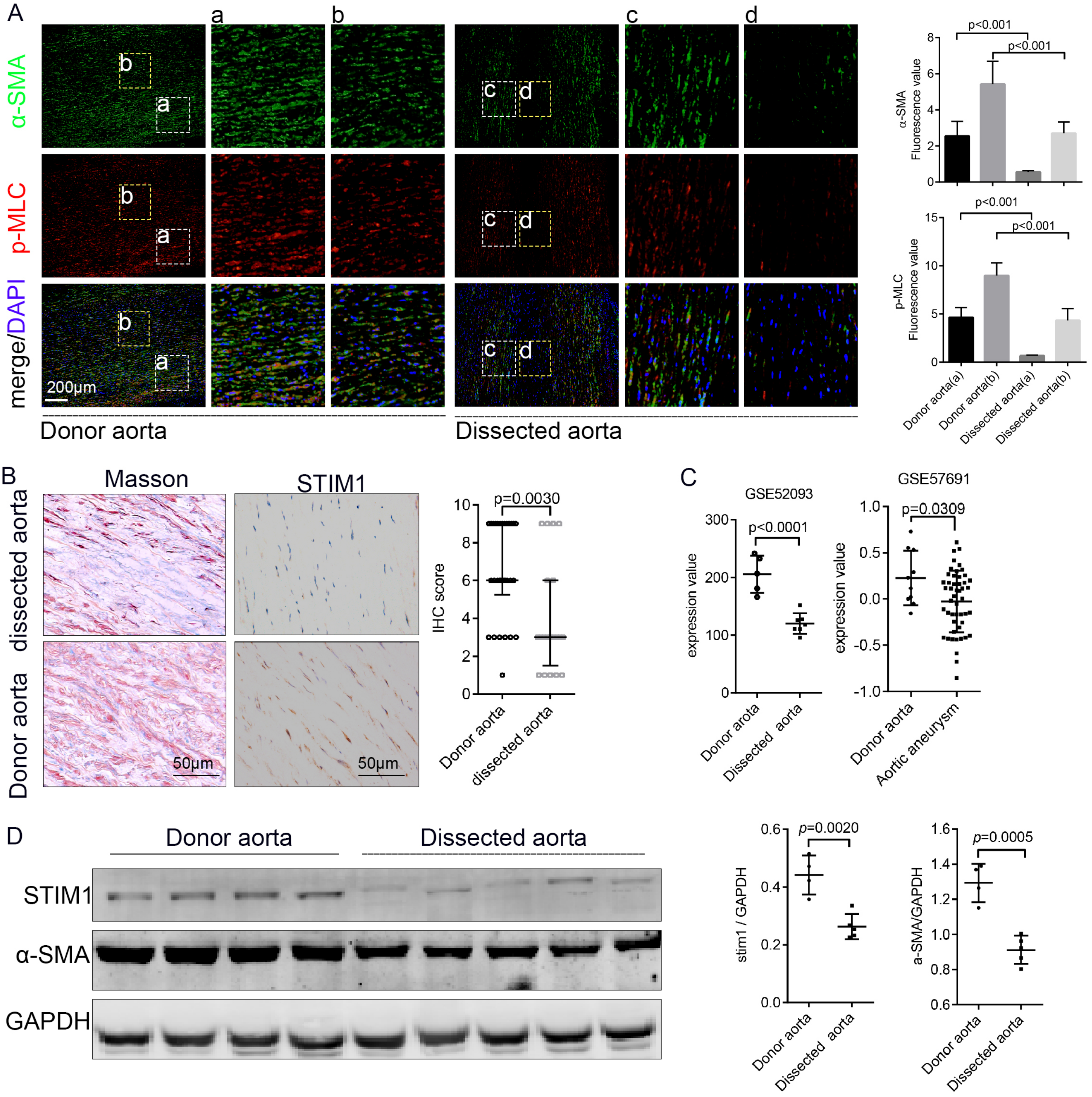
A
a
b
c
d
α-SMA
p-MLC
merge/DAPI
200μm
Donor aorta
Dissected aorta
p<0.001
p<0.001
α-SMA Fluorescence value
p-MLC Fluorescence value
Donor aorta(a)
Donor aorta(b)
Dissected aorta(a)
Dissected aorta(b)
B
Masson
STIM1
dissected aorta
Donor aorta
50μm
50μm
p=0.0030
IHC score
Donor aorta
dissected aorta
C
GSE52093
p<0.0001
expression value
Donor arota
Dissected aorta
GSE57691
p=0.0309
expression value
Donor aorta
Aortic aneurysm
D
Donor aorta
Dissected aorta
STIM1
α-SMA
GAPDH
p=0.0020
stim1 / GAPDH
Donor aorta
Dissected aorta
p=0.0005
a-SMA/GAPDH
Donor aorta
Dissected aorta

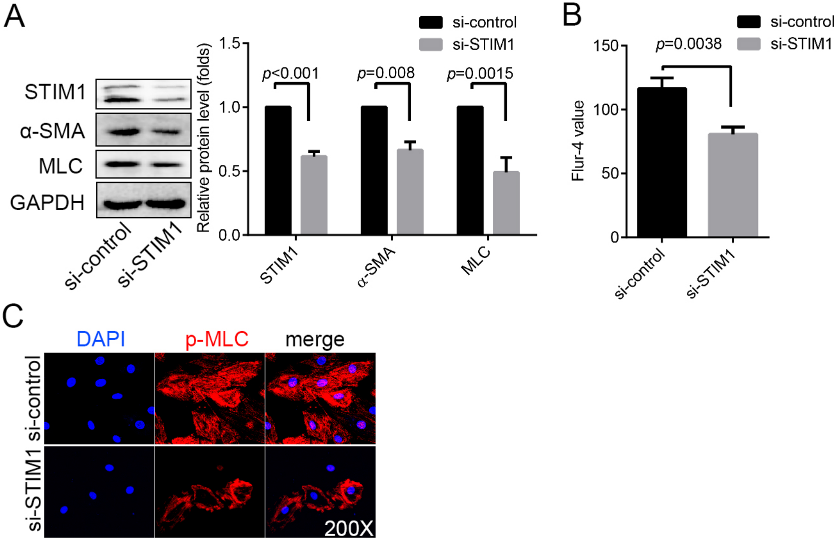
A
STIM1
α-SMA
MLC
GAPDH
si-control
si-STIM1
Relative protein level (folds)
1.5
1.0
0.5
0.0
p<0.001
p=0.008
p=0.0015
STIM1
α-SMA
MLC
B
si-control
si-STIM1
Flur-4 value
150
100
50
0
p=0.0038
si-control
si-STIM1
C
DAPI
p-MLC
merge
si-control
si-STIM1
200X

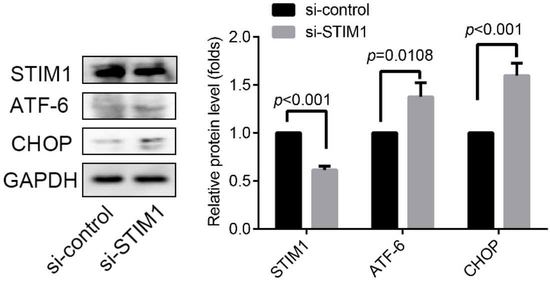
STIM1
ATF-6
CHOP
GAPDH
si-control
si-STIM1
si-control
si-STIM1
Relative protein level (folds)
2.0
1.5
1.0
0.5
0.0
p<0.001
p=0.0108
p<0.001
STIM1
ATF-6
CHOP

1. Clinical samples.

This study protocol was approved by the Clinical Research Ethics Committees of Renmin Hospital of Wuhan University of China, and informed written consent was obtained from each subject. Aortic specimens were collected from acute type A thoracic aortic dissection (TAD) patients (n=28), who underwent emergence aortic replacement surgery between, and donors of brain death patients (n=14). The clinical characters of these patients were recorded in detail and summarized (Table S1). Note: the samples and the summary of these patients were shared with another paper that under submitting.

Table S1. The clinical characters of the patients that contributed the sample in this study

	AD group(n=28)	Donor group(n=14)	P value
Male	19/28	13/14	<0.05
Age(mean±SD)	51.46±11.72	40.71±10.64	<0.05
Hypertension	21/28	6/14	0.403
Type 2 diabetes	1/28	2/14	0.240
Liver Complications	10/28	7/14	0.370
Renal Complication	11/28	4/14	0.384

2. siRNA transfection

H-ASMCs were plated in 6-wells plate. Transfection was performed with lipofectamine 2000 reagent (Invitrogen, CA, USA) according to the manufacturer's instructions when the culture cells reached 60% confluence.The cells were harvested 48h after transfection with STIM1-siRNA (sense,5’-GGCUCUGGAUACAGUGCUCdTdT-3’) or scrambled-siRNA, which were chemically synthesized (Ribobio company, Guangzhou, China).

3. The antibodies that used in this study were summarized and shown in Table S2

.

Table S2. The detail of antibodies used in this study

Antibody (target)	Company and catalog	Dilution		
		Western blotting	IHC	IF
GAPDH	Proteintech, 60004-1-Ig	1:2000	-	-
STIM1	Boster, PB0461	1:200	1:50	1:50
ACTA2(α-SMA)	Proteintech ,14395 -1-AP	1:800	-	1:200
ACTA2(α-SMA)	Boster, A01072	-	-	1:100
MLC	Proteintech, 10906-1-AP	1:500	-	-
p-MLC	CST, #3675	1:800	-	1:100
F-actin	Sangon biotech, E607503	-	-	1:1000
p-Smad2/3	CST, #8828	1:1000	-	-
Smad2/3	CST, #8685	1:1000	-	1:200
ATF-6	Proteintech, 24169-1-AP	1:500	-	-
CHOP	Proteintech, 15204-1-AP	1:1000	-	-
CY3 label goat anti-rabbit IgG(H+L)	Servicebio,CAT:G B21301	-	-	1:200
FITC label goat anti-rabbit IgG(H+L)	Servicebio,CAT:G B22303	-	-	1:100
CY3 label goat anti-mouse IgG(H+L)	Servicebio,CAT:G B21303	-	-	1:200
Anti-mouse IgG (H+L) (DyLight™800	CST, #5257	1:20000	-	-

4X PEG Conjugate)				
Anti-rabbit IgG (H+L) (DyLight™800 4X PEG Conjugate)	CST, #5151	1:20000	-	-

3. Re-analyzed the GEO data set.

In this study, we re-analyzed two micro-array data set of the clinical samples (*GEO:GSE52093; GEO: GSE57691*), and a set of establish aortic dissection model*(GEO: GSE107479)*. The data obtained by using GEO2R.
